# Causal Effects of Genetically Predicted Iron Status on Sepsis: A Two-Sample Bidirectional Mendelian Randomization Study

**DOI:** 10.3389/fnut.2021.747547

**Published:** 2021-11-19

**Authors:** Yuanlong Hu, Xiaomeng Cheng, Huaiyu Mao, Xianhai Chen, Yue Cui, Zhanjun Qiu

**Affiliations:** ^1^First Clinical Medical College, Shandong University of Traditional Chinese Medicine, Jinan, China; ^2^Department of Respiratory and Critical Care Medicine, The Affiliated Hospital of Shandong University of Traditional Chinese Medicine, Jinan, China; ^3^College of Traditional Chinese Medicine, Shandong University of Traditional Chinese Medicine, Jinan, China; ^4^Department of Emergency Medicine, The Second People's Hospital of Dongying, Dongying, China; ^5^Department of Dermatology, The Affiliated Hospital of Shandong University of Traditional Chinese Medicine, Jinan, China

**Keywords:** iron status, iron metabolism, sepsis, infection, bidirectional mendelian randomization analyses

## Abstract

**Background/Aim:** Several observational studies showed a significant association between elevated iron status biomarkers levels and sepsis with the unclear direction of causality. A two-sample bidirectional mendelian randomization (MR) study was designed to identify the causal direction between seven iron status traits and sepsis.

**Methods:** Seven iron status traits were studied, including serum iron, ferritin, transferrin saturation, transferrin, hemoglobin, erythrocyte count, and reticulocyte count. MR analysis was first performed to estimate the causal effect of iron status on the risk of sepsis and then performed in the opposite direction. The multiplicative random-effects and fixed-effects inverse-variance weighted, weighted median-based method and MR-Egger were applied. MR-Egger regression, MR pleiotropy residual sum and outlier (MR-PRESSO), and Cochran's *Q* statistic methods were used to assess heterogeneity and pleiotropy.

**Results:** Genetically predicted high levels of serum iron (OR = 1.21, 95%CI = 1.13–1.29, *p* = 3.16 × 10^−4^), ferritin (OR = 1.32, 95%CI = 1.07–1.62, *p* =0.009) and transferrin saturation (OR = 1.14, 95%CI = 1.06–1.23, *p* = 5.43 × 10^−4^) were associated with an increased risk of sepsis. No significant causal relationships between sepsis and other four iron status biomarkers were observed.

**Conclusions:** This present bidirectional MR analysis suggested the causal association of the high iron status with sepsis susceptibility, while the reverse causality hypothesis did not hold. The levels of transferrin, hemoglobin, erythrocytes, and reticulocytes were not significantly associated with sepsis. Further studies will be required to confirm the potential clinical value of such a prevention and treatment strategy.

## Introduction

Iron is considered an essential nutrient for both humans and pathogenic microbes. Iron limitation defends pathogenic microbes as a key form of innate immune, termed “nutritional immunity” (1, 2). Interestingly, iron overload-associated diseases, such as hereditary hemochromatosis and β-thalassemia, were also found increased infection susceptibility (1, 3). Recently, several MR studies also gave evidence of a significant effect of genetically high iron status on the susceptibility of skin and soft tissue infections.

Sepsis is defined as life-threatening organ dysfunction caused by a dysregulated host response to infection (4). Although knowledge about sepsis is growing all the time, it is a depressing fact that current efforts to develop new therapies have not been successful (5). Early antibiotic therapy was a widely accepted treatment for sepsis, which benefitted patients by improving survival prognosis (6–8). The above references suggest that early intervention is of great significance. The general consensus is that early identification of risk factors is essential for the early intervention of sepsis.

Several observational studies showed that patients with sepsis had significantly elevated levels of serum iron, ferritin, and transferrin saturation, but transferrin level was in the opposite direction (9–11). Nevertheless, the evidence from conventional observational studies always remains vulnerable to the threat of confounding and reverse causation. Thus, the direction of causality between iron status and sepsis remains unclear. As an epidemiological approach, mendelian randomization (MR) uses genetic variants as instrumental variables (IVs) to infer expose-outcome causation from observational data, which is closely analogous to the clinical randomized controlled trials (RCTs). This also presents an opportunity to promote drug repurposing (12). Based on Mendel's laws of independent assortment and segregation, each allele is randomly transmitted to the offspring with an equal probability, which is exploited as a way of randomizing participants into different levels of the iron status traits (13, 14). Besides, the unidirectional flow of biological information from the parental generation to progeny can limit the effects of reverse causation (15). Herein, we designed a two-sample bidirectional MR study to identify the direction of causal effects between seven iron status traits and sepsis, using available public data from a large-scale genome-wide association study (GWAS) for sepsis and two GWAS meta-analyses for iron status traits.

## Methods

### Study Design and Data Source

This study adhered to the STROBE-MR guidelines (16) and the key principles of the Strengthening the Reporting of Observational Studies in Epidemiology (STROBE) guidelines (17). We designed a two-sample bidirectional MR study to imply the direction of causality between iron status and sepsis, which involved two sets of analyses. MR analysis was first performed to estimate the causal effect of iron status on the risk of sepsis and then performed in the opposite direction.

We retrieved and downloaded the summary data of GWAS (Supplementary Table 1) for all exposures and outcomes from the IEU OpenGWAS Project (https://gwas.mrcieu.ac.uk/) (18). The genetic variations data associated with iron status traits (serum iron, log10 ferritin, transferrin saturation, and transferrin) were obtained from the Genetics of Iron Status Consortium (GISC), which were the results of GWAS meta-analysis (*n* = 23,986, European ancestry) from 11 cohorts (19). For the entire population from 11 cohorts, 55% were women and 45% were men, the average age was 46.89 (*SD* = 17.84). The summary-level GWAS data of the other three biomarkers (hemoglobin, erythrocyte count, and reticulocyte count) were available from a previous study, which was the re-analysis and meta-analysis result (*n* = 172,952, European ancestry) from three GWAS cohorts (20). Fifty-two percent were women and 48% were men. Hemoglobin concentration, erythrocyte count, and reticulocyte count were measured from the whole blood samples using clinical hematology analyzers by the fluorescence or/and impedance flow cytometry.

Summary-level data of GWAS for sepsis were obtained from a re-analysis study, in which the subjects included 10,154 sepsis cases and 452,764 controls from UK Biobank (21, 22). Women constitute 54% and men represent 46%. The median age of all participants was 58 years, and the median age of sepsis cases was 60 years. Sepsis was defined based on a previously published list of explicit International Classification of Disease (ICD)-9 and ICD-10 codes from the Global Burden of Disease (GBD) study (23). Relevant details of the above GWAS study were reported elsewhere (21, 22).

Genotype imputation and associated quality control procedures have been previously described (19–22). To control for population structure, all analyses were adjusted for sex, age, and study-specific covariates such as genetic principal components, as listed for each study in Supplementary Table 1.

### Genetic Instrumental Variable Selection

Genetic instrumental variables were selected using the TwoSampleMR R package (version 0.5.6). We selected single nucleotide polymorphisms (SNPs) with a strong association (*p* < 5 × 10^−8^) and independent inheritance (*R*^2^ < 0.005, 10 MB window) without any linkage disequilibrium (LD) from GWAS summary data of iron status or sepsis. The SNPs with LD were identified and excluded by a clumping algorithm based on the 1,000 genomes reference panel. We also removed the SNPs with palindrome allele (A/T or G/C) to prevent strand ambiguity issues.

### Mendelian Randomization Analysis

The multiplicative random-effects (RE) and fixed-effects (FE) inverse-variance weighted (IVW) were used to assess the causal associations between iron status and the risk of sepsis. The effect measures were the odds ratio (OR) of the risk of sepsis, which was normalized to one *SD* increment in each iron status biomarkers. In addition, we conducted weighted median (WM)-based method and MR-Egger statistical sensitivity analyses to ensure the robustness of pleiotropic IVs (24). A *p*-value < 0.05 was considered significant.

### Sensitivity Analysis

The concept of “pleiotropy” refers to a phenomenon that a single genetic variant could influence multiple traits, which violates the fundamental assumption of being a valid IV (25). Two strategies were used to address the issues of pleiotropy (24, 26). First, associations of SNPs with risk factors of known sepsis were assessed by searching the GWAS Catalog (https://www.ebi.ac.uk/gwas/) (27) and PubMed (https://pubmed.ncbi.nlm.nih.gov/). Second, a combination of the MR-Egger regression and MR pleiotropy residual sum and outlier (MR-PRESSO) methods were used to estimate the unknown directional pleiotropy. However, MR-Egger and MR-PRESSO analyses typically require more than 3 IVs (28). The MRPRESSO R package (version 1) was applied for MR-PRESSO analysis.

Cochran's *Q* statistic was used to assess the heterogeneity of the instrument variable. The random-effects IVW model was used if the substantial heterogeneity was significant (24). We also used leave-one-out plots for IVW estimates to confirm that the effects were not unduly influenced by outliers potentially representing pleiotropic pathways.

## Results

Independent SNPs were selected as genetic instrumental variables based on independent and LD analyses, including two serum iron-associated SNPs, three independent ferritin-associated SNPs, three transferrin saturation-associated SNPs, and eight transferrin-associated SNPs (Supplementary Tables 2, 3).

**Figure 1:**
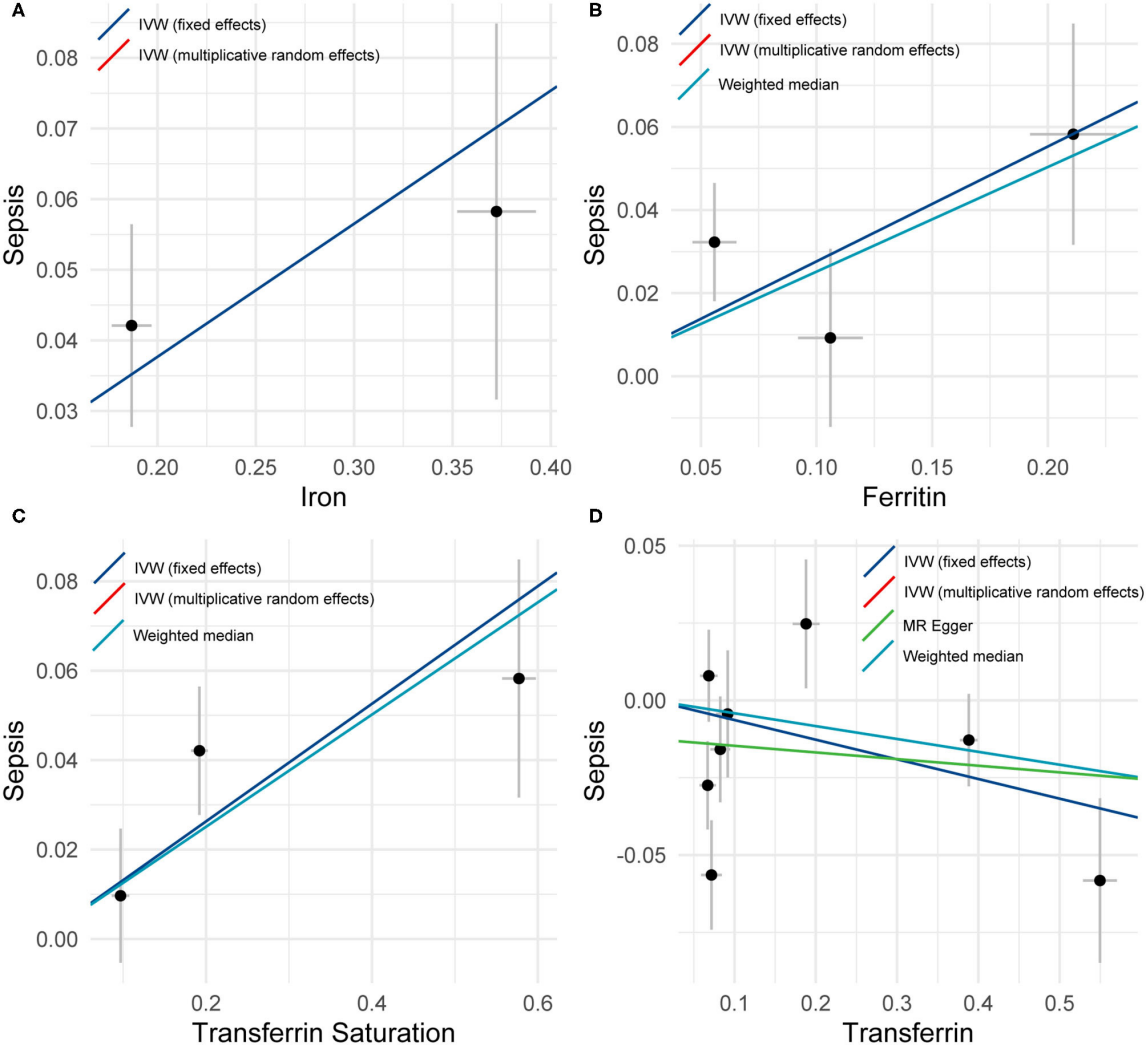
A plot showing the effect sizes of the single nucleotide polymorphism (SNP)-sepsis associations (y-axis), **(A)** the SNP-iron association (x-axis), **(B)** the SNP-ferritin (x-axis), **(C)** transferrin saturation (x-axis), and **(D)** transferrin (x-axis) with standard error bars. The slopes of the lines correspond to causal estimates using fixed-effects IVW (blue line), random-effects IVW (red line), MR-Egger (green line), and weighted median (light blue line) methods.

The primary MR analyses by fixed-effects IVW showed a significant causal effect of serum iron, ferritin, and transferrin saturation on the risk of sepsis (Table 1 and Figure 1). The results showed that the genetically predicted high levels of serum iron (OR = 1.21, 95%CI = 1.13–1.29, *p* = 3.16 × 10^−4^), ferritin (OR = 1.32, 95%CI = 1.07–1.62, *p* = 0.009), and transferrin saturation (OR = 1.14, 95%CI = 1.06–1.23, *p* = 5.43 × 10^−4^) were associated with an increased risk of sepsis. Results were consistent across random effect IVW and WM (Table 1). The above results were consistent and robust in all sensitivity analyses, and no heterogeneity and pleiotropy were observed (Table 1).

**Table 1:** Mendelian randomization estimates for the causal effect of iron status biomarkers on sepsis risk.

**Exposure**	**Number of SNPs**	**Methods**	**Parameter**	**OR or odds (95%CI)**	***P*-value**	**Cochran's *Q* statistic (*P*-value)**
Serum iron	2					
		IVW-FE	OR	1.21 (1.13, 1.29)	3.16E-04^***^	0.43 (0.511)
		IVW-RE	OR	1.21 (1.09, 1.34)	4.33E-08^***^	
Ferritin	3					
		IVW-FE	OR	1.32 (1.07, 1.62)	0.009^**^	2.27 (0.320)
		IVW-RE	OR	1.32 (1.09, 1.60)	0.009^**^	
		WM	OR	1.29 (1.03, 1.60)	0.023^*^	
Transferrin saturation	3					
		IVW-FE	OR	1.14 (1.06, 1.23)	5.43E-04^***^	1.86 (0.395)
		IVW-RE	OR	1.14 (1.06, 1.23)	3.36E-04^***^	
		WM	OR	1.13 (1.05, 1.23)	0.003^**^	
Transferrin	8					
		IVW-FE	OR	0.94 (0.86, 1.02)	0.023^*^	16.85 (0.018^*^)
		IVW-RE	OR	0.94 (0.89, 0.99)	0.142	
		WM	OR	0.96 (0.90, 1.02)	0.212	
		MR-Egger	OR	0.98 (0.86, 1.11)	0.753	14.94 (0.021^*^)
		MR-Egger	Intercept (odds)	−0.01 (-0.04, 0.02)	0.415	

*OR, odds ratio; CI, confidence interval; IVW, inverse variance weighted; RE, random-effects; FE, fixed-effects; WM, weighted median*.

* *< 0.05*;

** *<0.01*;

*** *<0.001*.

Conversely, low transferrin levels were significantly associated with increased risk of sepsis (OR = 0.94, 95%CI = 0.86–1.02, *p* = 0.023). Nevertheless, substantial heterogeneity among estimates of individual SNPs was detected in the analysis of Transferrin levels (IVW, Cochran's *Q* Statistic = 16.85, *I*^2^ = 58.45%, *p* = 0.018; MR-Egger, Cochran's *Q* Statistic = 14.94, *I*^2^ = 59.83%, *p* = 0.021). Thus, the random effect IVW model was employed, of which the results showed no significant association (OR = 0.94, 95%CI = 0.89–0.99, *p* = 0.142).

**Figure 2:**
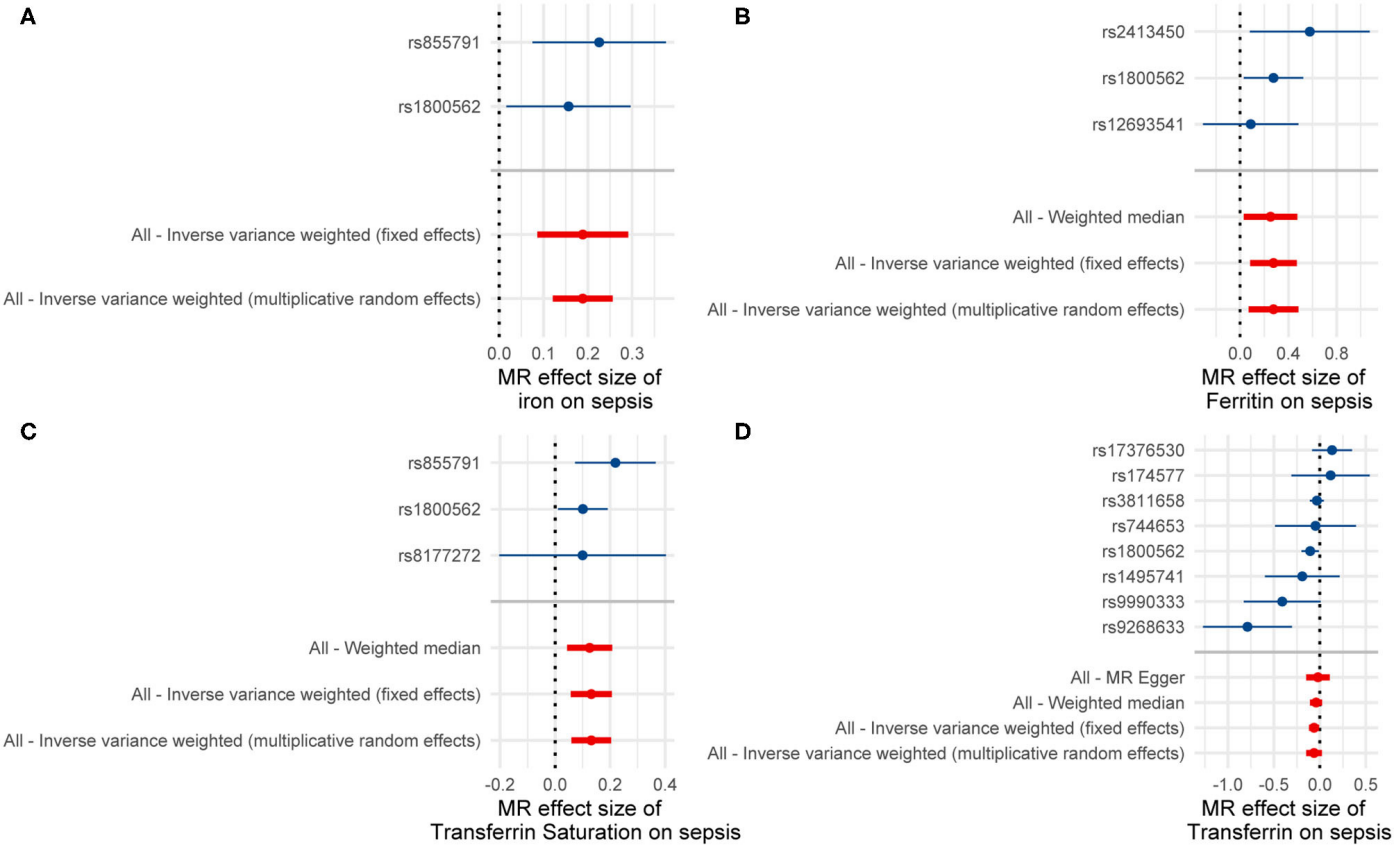
Results of the single and multi-SNP analyses for the SNP effect of **(A)** serum iron, **(B)** Ferritin, **(C)** transferrin saturation, and **(D)** transferrin on sepsis.

Single-SNP MR analyses showed that SNPs rs1800562 (HFE) and rs855791 (TMPRSS6) were associated with a significant effect of serum iron or transferrin saturation on sepsis, while SNPs rs1800562 (HFE) and rs2413450 (TMPRSS6) were significantly related to the causal effect of ferritin on sepsis (Figure 2). Using the leave-one-out analyses, we found that one independent SNP (rs1800562) could drive a significant effect of ferritin on sepsis. The significant associations of ferritin and sepsis did not remain after removing rs1800562 (Supplementary Figure 1).

The second set of MR analyses aimed to assess potential causal effects of sepsis on iron status (Supplementary Tables 4, 5). Results showed no significant causal relationship between sepsis and iron status (Table 2 and Supplementary Figure 2). As described in Table 2, we observed high statistical heterogeneity in the fixed-effects IVW MR analysis of transferrin saturation and transferrin, therefore, the random-effect IVW model was adopted as a sensitivity analysis, of which the results still showed no significant association.

**Table 2:** Mendelian randomization estimates for the causal effect of sepsis on iron status biomarkers.

**Outcome**	**Number of SNPs**	**Methods**	**Parameter**	**β or odds (95%CI)**	***P*-value**	**Cochran's *Q* statistic (*P*-value)**
Serum iron	4					
		IVW (FE)	β	−0.02 (−0.15, 0.12)	0.832	5.56 (0.135)
		IVW (RE)	β	−0.02 (−0.2, 0.17)	0.876	
		WM	β	−0.11 (−0.29, 0.07)	0.257	
		MR Egger	β	1.43 (0.22, 2.64)	0.147	0.01 (0.996)
		MR Egger	Intercept (Odds)	−0.12 (−0.22, −0.02)	0.143	
Ferritin	3					
		IVW (FE)	β	0 (−0.15, 0.14)	0.953	0.46 (0.796)
		IVW (RE)	β	0 (−0.07, 0.06)	0.902	
		WM	β	0.01 (−0.15, 0.18)	0.883	
Transferrin saturation	3					
		IVW (FE)	β	−0.01 (−0.16, 0.15)	0.936	7.69 (0.021^*^)
		IVW (RE)	β	−0.01 (−0.31, 0.3)	0.968	
		WM	β	0.11 (−0.1, 0.32)	0.302	
Transferrin	3					
		IVW (FE)	β	−0.02 (−0.18, 0.14)	0.819	18.89 (7.91E-05^***^)
		IVW (RE)	β	−0.02 (−0.51, 0.47)	0.941	
		WM	β	0.07 (−0.2, 0.34)	0.603	

*CI, confidence interval; IVW, inverse variance weighted; RE, random-effects; FE, fixed-effects; WM, weighted median*.

* *< 0.05*;

*** *< 0.001*.

Iron is needed mainly for hemoglobin and erythrocytes production, therefore, we wondered whether the levels of hemoglobin, erythrocytes, and reticulocytes could affect the risk of sepsis and whether sepsis could influence the level of this biomarker. The results of MR analysis suggested no statistically significant effects on whether the sepsis served as exposure or outcome, which agreed with the results of sensitivity analyses (Supplementary Figures 3, 7 and Supplementary Table 6). A random-effects model was applied to estimate the causal effects of sepsis on the above three biomarkers due to the high heterogeneity level. Moreover, no pleiotropy was found in all analyses.

## Discussion

In this bidirectional MR study, we investigated the association of iron status biomarkers with the risk of sepsis, of which genetic variants that proxy the effect were identified from publicly available large-scale GWAS data. Our findings showed evidence that genetic predisposition to higher levels of serum iron, ferritin, and transferrin saturation was causally associated with a higher risk of sepsis. Genetic predisposition to sepsis did not appear to influence any of the studied iron status markers concentration (serum iron, ferritin, transferrin saturation, and transferrin).

In contrast with the previous MR studies of iron status (29, 30), the number of IVs in our study was more conservative. Previous research confirmed that rs1800562 and rs855791 were the primary determinants of variation associated with HFE and TMPRSS6 in serum iron status (31, 32). Searching the GWAS Catalog and PubMed, we found that rs1800562 and rs1495741 were associated with low-density lipoprotein cholesterol (LDL-C), total cholesterol (TC), or triglyceride (30, 33), but there is currently no evidence to confirm the above lipid metabolism biomarkers as risk factors for sepsis susceptibility (34, 35).

The results from this MR study were fundamentally consistent with recent observational studies (9–11), except for transferrin. Increased ferritin and serum iron concentrations were also associated with greater severity (36) and a poor prognosis (37, 38) for sepsis. As an important form of host defense, nutritional immunity presents with limiting the availability of iron to pathogens (1, 39, 40). One such example is anemia of inflammation (41), but it does not contradict our idea that iron status parameters were causally positively associated with the risk of sepsis. High iron status may enhance susceptibility for sepsis *via* dysregulated host-pathogens interaction (39), which is a disorder of iron homeostasis. An important fact from the perspective of pathogens is that iron accumulation favors the growth and survival of the pathogens on the host (42–44). Besides, from the host perspective, high iron status could affect the innate immune functions and promotes the synthesis and release of inflammatory factors (2, 3, 45). The evidence of pre-clinical studies supported iron chelators as a potential therapeutic agent of sepsis (46–49). Therefore, targeting high iron status was a potential therapeutics and promising direction for the prevention and treatment of sepsis.

There are several limitations that we could not overlook in this study. First, population limited to European ancestry hampered the generalization of findings to individuals of other ancestries. Second, we could not determine whether there were non-linear between iron status biomarkers levels and the risk of sepsis, such as threshold effect or *U*-shaped relationship. Third, this study did not adequately investigate all indicators for the assessment of human iron status traits, such as hemoglobin and transferrin receptors.

In summary, this present bidirectional MR analysis suggested the causal association of high iron status with an increased risk of sepsis, while the reverse causality hypothesis did not hold. The levels of transferrin, hemoglobin, erythrocytes, and reticulocytes were not significantly associated with sepsis. Future studies are needed to explore the exact mechanism and confirm the potential clinical value of such a prevention and treatment strategy.

## Data Availability Statement

This data can be found at: Publicly available datasets were analyzed in this MR study. GWAS summary-level datasets were downloaded from IEU OpenGWAS Project (https://gwas.mrcieu.ac.uk/), including serum iron (GWAS ID: ieu-a-1049), transferrin (GWAS ID: ieu-a-1052), transferrin saturation (GWAS ID: ieu-a-1051), ferritin (GWAS ID: ieu-a-1050), hemoglobin (GWAS ID: ebi-a-GCST004615), erythrocytes count (GWAS ID: ebi-a-GCST004601), reticulocytes count (GWAS ID: ebi-a-GCST004622), and sepsis (GWAS ID: ieu-b-69).

## Author Contributions

All authors contributed to the design, interpretation, and writing of the paper.

## Funding

This work was supported by Shandong Provincial Natural Science Foundation, China (ZR2020MH392), Shandong Medical and Health Technology Development Project (2018WS193), and Qilu Wellness and Health Leading Talent Training Project in 2020.

## Conflict of Interest

The authors declare that the research was conducted in the absence of any commercial or financial relationships that could be construed as a potential conflict of interest.

## Publisher's Note

All claims expressed in this article are solely those of the authors and do not necessarily represent those of their affiliated organizations, or those of the publisher, the editors and the reviewers. Any product that may be evaluated in this article, or claim that may be made by its manufacturer, is not guaranteed or endorsed by the publisher.
